# 92R Monoclonal Antibody Inhibits Human CCR9^+^ Leukemia Cells Growth in NSG Mice Xenografts

**DOI:** 10.3389/fimmu.2018.00077

**Published:** 2018-01-29

**Authors:** Beatriz Somovilla-Crespo, Maria Teresa Martín Monzón, Maria Vela, Isabel Corraliza-Gorjón, Silvia Santamaria, Jose A. Garcia-Sanz, Leonor Kremer

**Affiliations:** ^1^Department of Immunology and Oncology, Centro Nacional de Biotecnologia (CNB-CSIC), Madrid, Spain; ^2^Protein Tools Unit, Centro Nacional de Biotecnologia (CNB-CSIC), Madrid, Spain; ^3^Department of Cellular and Molecular Medicine, Centro de Investigaciones Biologicas (CIB-CSIC), Madrid, Spain

**Keywords:** cancer, therapeutic antibodies, combinations, oncology, chemokine receptors

## Abstract

CCR9 is as an interesting target for the treatment of human CCR9^+^-T cell acute lymphoblastic leukemia, since its expression is limited to immature cells in the thymus, infiltrating leukocytes in the small intestine and a small fraction of mature circulating T lymphocytes. 92R, a new mouse mAb (IgG2a isotype), was raised using the A-isoform of hCCR9 as immunogen. Its initial characterization demonstrates that binds with high affinity to the CCR9 N-terminal domain, competing with the previously described 91R mAb for receptor binding. 92R inhibits human CCR9^+^ tumor growth in T and B-cell deficient Rag2^−/−^ mice. *In vitro* assays suggested complement-dependent cytotoxicity and antibody-dependent cell-mediated cytotoxicity as possible *in vivo* mechanisms of action. Unexpectedly, 92R strongly inhibited tumor growth also in a model with compromised NK and complement activities, suggesting that other mechanisms, including phagocytosis or apoptosis, might also be playing a role on 92R-mediated tumor elimination. Taken together, these data contribute to strengthen the hypothesis of the immune system’s opportunistic nature.

## Introduction

Chemokine receptors and their ligands are crucial for organogenesis and lymphocyte trafficking, both in homeostasis and inflammation (1). The chemokine receptor 9 (CCR9) expression in normal cells is limited to immature T lymphocytes in the thymus (2–5), small bowel infiltrating cells (6), a small fraction of circulating memory T lymphocytes (CCR9^+α^4β7^high^) (7), IgA secreting plasma B cells (1), and plasmacytoid dendritic cells (8). Up to now, the only known ligand for CCR9 is the chemokine CCL25 (3, 9). CCL25 is secreted in the thymus by epithelial and dendritic cells (4, 10) and also by the small intestinal crypt epithelium (6). The CCL25–CCR9 interaction controls migration of thymocytes within the thymus and homing of mature CCR9^+^ lymphocytes to the intestinal tract (7). In addition, there is a strong association between aberrant chemokine receptor expression on tumor cells (i.e., CXCR4 or CCR7) with cancer progression, poor prognosis, and organ-selective metastases (11–13). For CCR9 expression in tumor cells, the data are still limited, but CCR9 expression correlates with the ability of the tumor to generate metastasis in the small intestine (14–16), the main site, in addition to the thymus, of CCL25 secretion. CCR9 overexpression in acute and chronic T cell leukemia has been linked to disease aggressiveness (17). In addition, aberrant CCR9 expression in prostate tumors, breast cancer, or melanoma, has been correlated with *in vitro* invasiveness in response to CCL25 (14, 15, 17–23). Tumor cells-expressing CCR9 have competitive advantages, since engagement of the CCL25 ligand enhances cell survival and provides resistance to apoptosis *via* the phosphatidylinositide 3-kinase/Akt pathway on several solid tumors (20, 21, 24–30); it activates the JNK1 antiapoptotic pathway in leukemic cells (31) and participates in Notch1-mediated cell proliferation (19).

Targeted therapies and immunotherapy have safety advantages over non-specific cytotoxic agents, since they are able to discriminate between normal and tumor cells. Therefore, their use for the treatment of cancer is in constant expansion (32). The described therapeutic tools that specifically target human CCR9^+^-tumors and have been tried in xenogeneic models are limited to the use of the CCR9-ligand coupled to a cytotoxic agent (CCL25-PE38 fusion protein) (33), the use of ligand-specific antibodies, alone or in combination with etoposide (25), or the mAb 91R that selectively inhibited growth of a human acute T lymphoblastic leukemia (T-ALL) cell line in Rag2^−/−^ xenografts (34). The first two strategies eliminate tumor cells by targeting the CCL25–CCR9 interaction, whereas the last directly targets the cells expressing CCR9. These data provide evidence of CCR9 as a potential target for cancer immunotherapy.

With the aim of selecting other anti-CCR9 mAb with (i) different specificities, (ii) different affinities for CCR9, (iii) provided of different mechanism(s) of action, and (iv) displaying high melting points, new hybridomas were generated and screened. mAbs with these properties could be more convenient to be used for therapeutic purposes. Here, we report the generation and characterization of 92R, an anti-CCR9 mAb able to selectively inhibit *in vivo* growth of human acute T-ALL cells transplanted into immunodeficient Rag2^−/−^ or NSG mice. This antibody has therapeutic potential for the targeted elimination of CCR9^+^-tumor cells, used either alone or in combination with other therapies.

## Materials and Methods

### Cells and Reagents

Human embryonic kidney 293 (HEK-293, CRL-1573) cells and HEK-293 cells stably transfected with the human chemokine receptor CCR9, or the empty vector (pCIneo) were a kind gift of A. Zaballos (CNB-CSIC, Madrid, Spain), cells were cultured as described (3). MOLT-4 (CRL-182) and Jurkat (TIB-152) human T-ALL cell lines were obtained from the American Type Culture Collection (ATCC). Cells were cultured in Dulbecco’s modified Eagle’s medium (Gibco) supplemented with 10% fetal bovine serum (FBS, Gibco), 2 mM l-glutamine, 50 U/ml penicillin, and 50 µg/ml streptomycin (complete medium). Neomycin-resistant stable HEK-293 transfectants were cultured in the presence of 1 mg/ml G418 (Sigma) and periodically tested for CCR9 expression (not shown). Recombinant human CCL25 and CXCL12 were purchased from Peprotech. We used the following antibodies: 3C3 (ATCC HB-12653), 112509, mouse mAb anti-hCCR9 (IgG2a; R&D) and M4, a serum pool generated by immunizing BALB/c mice with three intraperitoneal injections of 10^7^ MOLT-4 cells in PBS (days 1, 25, and 50); sera were collected on day 60.

### Generation of Human CCR9-Specific mAb

Murine 91R and 92R anti-human CCR9 mAb were raised after immunization of BALB/c mice with a gene gun (Bio-Rad) particle-mediated DNA administration of the pCIneo plasmid bearing the human CCR9 cDNA, as previously described (34). Mouse sera were collected 7–10 days (d) after the last boost and tested for specific antibodies by flow cytometry using stably transfected hCCR9-HEK-293 cells, and pCIneo-HEK-293 cells as negative control. Selected mice were boosted intravenously with 10^7^ hCCR9-HEK293 cells 3 and 2 days prior to splenocyte fusion (35). Two weeks post-fusion, culture supernatants were screened by flow cytometry for CCR9-specific antibodies using hCCR9-HEK293 cells. Positive hybridomas were cloned, mAb purified from culture supernatants and antibody isotype determined by enzyme-linked immunosorbent assay (ELISA) (35).

### Flow Cytometry

For staining, 2 × 10^5^ cells/well were centrifuged in V-bottom 96-well plates and washed with phosphate-buffered saline, pH 7.4 (PBS) supplemented with 0.5% bovine serum albumin (BSA), 1% FBS, and 0.1% sodium azide (PBSst). Non-specific binding of the mAb to the cell surface was blocked by preincubating the cells with 40 µg/ml rat IgG (Sigma) in a 100 µl final volume (20 min, 4°C). Cells were incubated with the primary mAb (30 min, 4°C), washed, and the binding was revealed with a secondary FITC- or PE-goat F(ab’)2 anti-mouse IgG (H + L) antibody (Beckman Coulter; 30 min, 4°C). Samples were analyzed on an Epics XL or a Cytomics cytometer (Beckman Coulter). For competition analyses, cells were incubated with 50 µl of either the unlabeled antibody or an isotype-matched mAb (10 µg/ml, 40 min, 4°C), followed by 50 µl of an anti-CCR9 biotin-labeled antibody (0.5–2 µg/ml, 30 min, 4°C). After washing, FITC- or PE-conjugated streptavidin was added (30 min, 4°C). Cell staining was evaluated by flow cytometry.

### Competitive ELISA

Microtiter plates (Maxi-sorb, Nunc) were used to coat the hCCR9(2–22) synthetic peptide (1 µg/ml in PBS), overnight at 4°C. Afterward, the unoccupied protein-binding sites in the wells were blocked with 0.5% BSA in PBS. Previously titrated biotin-labeled mAb was mixed with unlabeled mAb (2 µg/ml in PBS–0.5% BSA), added to the plate and incubated 1 h, at room temperature. Antibodies bound to the plate were detected with peroxidase-labeled streptavidin (Sigma) and revealed with o-phenylenediamine dihydrochloride (4 mg/ml in 0.15 M sodium citrate buffer, pH 5.0; Sigma). The reaction was stopped with 3N sulfuric acid and the O.D. 492 nm determined. Antibodies were biotinylated with Hydrazide-LC-Biotin (ThermoFisher Scientific) following the supplier’s instructions.

### Chemotaxis Assays

Migration assays were performed in transwell inserts (Costar) with a 5-µm pore diameter. MOLT-4 cells were re-suspended in RPMI with 1% BSA and 25 mM HEPES, pH 7.4 (10^7^ cells/ml), and 100 µl aliquots were loaded into the upper inserts. Samples of 0–300 nM human CCL25, prepared in 600 µl of the same medium, were placed in the lower wells. After 2 h incubation at 37°C, 5% CO_2_, inserts were removed and the number of cells that had migrated from the transwell insert to the well were counted on an EPICS XL flow cytometer. To analyze the antibody-blocking activity of CCL25-induced migration, MOLT-4 cells were preincubated with different amounts of anti-CCR9 mAb or irrelevant isotype-matched mAb before being loaded into the transwell. For these experiments, 200 nM human CCL25 was used as chemoattractant.

### Peptide Synthesis

Linear peptides were synthesized in the Proteomic facility of the CNB, with an automated multiple-peptide synthesizer (AMS 422, Abimed) using a solid-phase procedure and standard Fmoc-chemistry. The synthesis of multiple peptides was performed simultaneously, on a cellulose membrane, by sequential conjugation of membrane-protected amino acids (aa), from their carboxy terminal ends. The application of the activated aa was carried out using the Auto-Spot Robot (ASP222, Abimed) (36). Two independent membranes were prepared with the same set of peptides (each 12 aa long, 10 aa overlap), spanning the complete hCCR9-A isoform (369 aa). In addition, a similar synthesis was carried out where to peptides corresponding to aa 8–19 of hCCR9-A, aa in positions 11–16, each one of them was substituted for each of the remaining proteinogenic aa. Membranes were blocked by incubation with 1% BSA in PBS for 1 h at room temperature, washed, and then incubated for 2 h with anti-CCR9 or isotype control antibodies (in PBS containing 1% BSA and 0.05% Tween-20). After three additional washes, membranes were incubated with a peroxidase-labeled goat anti-mouse IgG antibody (Sigma), for 1 h at room temperature, developed with a chemiluminescence system (GE Healthcare), and exposed to standard X-ray film. The densitometric quantification of the signal obtained at each spot was performed with the ImageJ software.

### Surface Plasmon Resonance Analyses

Surface Plasmon Resonance experiments were carried out in a biosensor Biacore 3000 (Biacore, GE Healthcare), using HBS-EP (10 mM HEPES, 0.15 M NaCl, 3 mM EDTA, 0.005% Surfactant P20, pH 7.4) as running buffer. At the end of each binding cycle, the sensor surface was regenerated with 10 mM glycine–HCl, pH 1.5, allowing resonance signals to return to baseline values. A synthetic peptide corresponding to the aa in positions 2–22 of the human CCR9 isoform A (hCCR9A, identifier: P51686-1), with an additional cystein residue in its N terminus (C-TPTDFTSPIPNMADDYGSEST) was immobilized on a carboxymethylated dextran CM5 sensor chip by an amine coupling reaction, as recommended by the supplier. A reference surface was generated in the same manner, except that all carboxyl groups were blocked in the absence of ligand. For kinetic analyses, immobilized antigen at low density was used to minimize mass transport effects and analyte rebinding. Anti-CCR9 mAbs were used as soluble analytes in HBS-EP buffer at concentrations ranking from 0.41 to 33 nM. The interaction analyses were carried out at 25°C with a flow rate of 30 µl/min. Data were collected for 90 s of association and 180 s of dissociation. For competition analyses, a biotin-labeled peptide corresponding to aa 2–19 of hCCR9A (biotin-K-TPTDFTSPIPNMADDYGS) was captured on a streptavidin-coated chip (SA). Anti-CCR9 mAbs at a constant concentration (10 nM) were mixed with competitor synthetic peptides at different concentrations (0.01–1 µM). The antibody–peptide mixtures were preincubated for 30 min before they were injected into the biosensor. Sensograms were overlaid, aligned, and analyzed with BIA evaluation Software 4.1. The *K*_D_ was determined by fitting the data using a bivalent analyte model. All data set were processed using a double-referencing method (37).

### Xenograft Assays

BALB/c Rag2^−/−^ mice (Taconic Bioscience) were bred in the CNB animal facility and used at ages ranging from 8 to 22 weeks. For *in vivo* experiments, MOLT-4 cells (2 × 10^6^) were inoculated sub-cutaneously (s.c.) in the flank of Rag2^−/−^ mice on day 0. In these experimental conditions, 80–90% of the cell inoculations gave rise to tumors. The animals carrying the MOLT-4 cells were divided into four groups, which were inoculated intraperitoneally (i.p.) with anti-hCCR9 91R, its isotype control (IgG2b), anti-hCCR9 92R, or its isotype control (IgG2a) on days 1, 8, 15, and 22 (4 mg/kg on day 1 and 8; 2 mg/kg on days 15 and 22). Tumor size was measured with a Vernier caliper (Mitutoyo) and tumor volume (mm^3^) calculated as *V* = [axial diameter length, mm] × [(rotational diameter, mm)^2^/2]. Mice were sacrificed and tumors were weighted and processed for histology. Tumor burden is expressed as percent tumor weight relative to that of isotype control-treated mice.

NOD.Cg-*Prkdc^scid^ Il2rg^tm1Wjl^*/SzJ stock # 005557 (NSG mice, Jackson Laboratories, ME, USA) were bred in the CIB animal facility and used for similar experiments as described in the above paragraph. Animals were injected subcutaneously on day 0 with MOLT-4 cells (1 × 10^6^) in the flank. Two groups were inoculated intra-peritoneally with 4 mg/kg on day 2 and day 7 with anti-hCCR9 92R or its isotype control (IgG2b). All mice were sacrificed at the same time and tumors were weighted and processed for histology.

### Complement-Dependent Cytotoxicity (CDC)

MOLT-4 cells (10^5^ cells in 100 µl) were plated on each well of a 96-well V-bottom plate. The cells were incubated with the indicated concentrations of 92R, 91R (anti-hCCR9) or isotype-matched control mAb (30 min, 37°C), centrifuged, and washed. Active or 56°C heat-inactivated baby rabbit complement (25%; AbD Serotec) was added in serum-free Dulbecco’s modified Eagle’s medium with 1% BSA (1 h, 37°C). The complement in M4 serum was also heat inactivated (56°C, 30 min). The number of non-viable cells was evaluated by flow cytometry after staining the cells with the viability exclusion marker 7-aminoactinomycin (7-AAD; BD Biosciences; 10 min, 4°C); each condition was analyzed in triplicate. Specific lysis was calculated as: 100 × (% dead cells with active complement − % dead cells with inactive complement)/(100% − % dead cells with inactive complement).

### Antibody-Dependent Cell-Mediated Cytotoxicity (ADCC)

Murine NK cells were isolated from spleens from BALB/c mice using the Auto Macs Pro negative selection system (Miltenyi). After purification, cells were analyzed for mCD3, mCD45 and mCD49b expression by flow cytometry. The purity of NK cells, defined as CD3^−^CD45^+^CD49b^+^, in all the preparations was at least 90%. Cells were cultured for 6–7 days in RPMI 1640 (Lonza), supplemented with 10% FBS, and 1,000 U/ml murine recombinant IL-2 (Peprotech). For cytotoxicity assays, target MOLT-4 cells labeled with Cell Trace CFSE (Invitrogen) were preincubated (30 min) with the indicated mAb concentrations. NK and target cells were cocultured (4 h) at a 20:1 ratio in RPMI–10% FBS, then stained with 7-AAD (10 min, 4°C) and analyzed by flow cytometry. Gating on 7-AAD-positive cells within the CFSE^+^ population indicated the proportion of dead target cells. Specific killing was calculated as: 100 × [(% dead target cells in sample − % spontaneous dead target cells)/(100 − % spontaneous dead target cells)]. Target cells incubated without effector cells were used to assess spontaneous cell death.

### Statistical Analyses

Statistical analyses were performed using GraphPad Prism 4 software. Statistical significance was established at *P* < 0.05 as evaluated by Student’s *t-*test, unless otherwise indicated. Results are shown as mean ± SEM.

## Results

### Initial Characterization of the Anti-hCCR9 Chemokine Receptor mAb 92R

**Figure 1:**
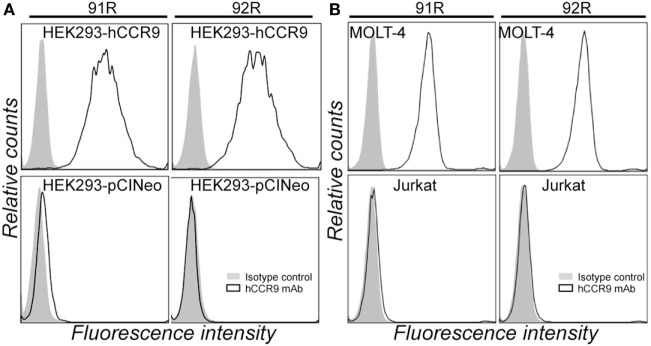
92R mAb identifies human chemokine receptor CCR9. **(A)** Representative flow cytometry staining of HEK-293 cells stably transfected with either pCIneo-hCCR9 or the empty pCIneo vector using the mAbs 91R, 92R (empty histograms), or isotype-matched control mAbs (IgG2b and IgG2a, respectively) (filled histograms). **(B)** MOLT-4 and Jurkat human leukemia cells were stained with the anti-human CCR9 mAb 91R and 92R (empty histograms) or isotype-matched control mAbs (filled histograms) and analyzed by flow cytometry.

The murine anti-hCCR9 mAb 92R (IgG2a) was generated after gene gun immunization with the full-length hCCR9-A cDNA coding sequence, inserted in a eukaryotic expression vector (pCINeo), using the same strategy as described for 91R mAb (34). Specificity of the binding of 92R to hCCR9-A protein was assessed by flow cytometry analyses of HEK-293 cells stably transfected with the construct used for immunization, using as negative control the same cells transfected with the empty pCINeo plasmid (Figure 1A). In addition, we demonstrated that 92R, similarly to 91R also stained cells expressing the endogenous CCR9 protein in the MOLT-4 T-ALL cell line, but failed to stain the Jurkat T-ALL cells that do not express CCR9 on their cell surface (Figure 1B).

**Figure 2:**
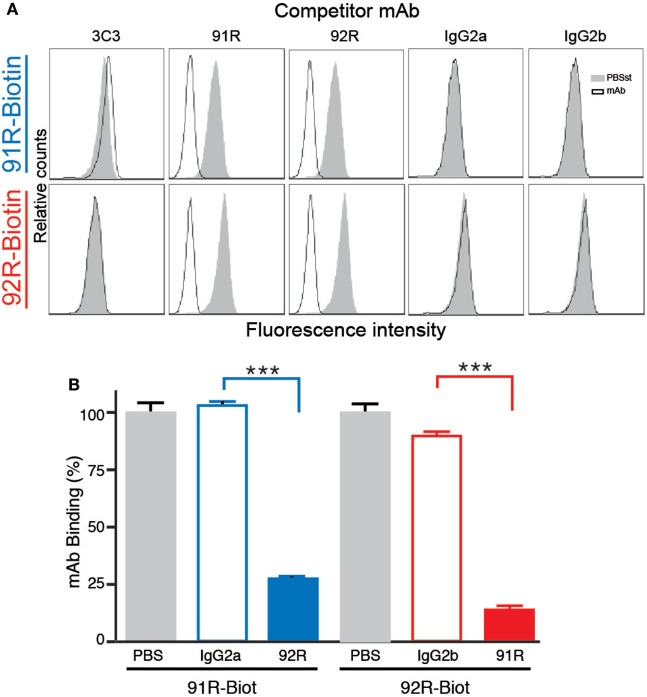
92R and 91R mAbs compete with each other for binding to hCCR9. **(A)** Competitive binding analyses to CCR9^+^ MOLT-4 cells. Cells were preincubated with unlabeled 3C3, 91R, 92R anti-CCR9 mAbs, or their isotype controls (IgG2a or IgG2b) and, without washing the antibody excess, stained with either biotinylated 91R (top row) or biotinylated 92R (bottom row). After washing, binding of the biotinylated antibodies to the MOLT-4 cells was revealed with streptavidin-FITC and analyzed by flow cytometry. **(B)** Enzyme-linked immunosorbent assay competitive binding analysis of anti-CCR9 mAbs to the hCCR9 (aa 2–22) peptide using biotin-labeled 91R and 92R in the presence of unlabeled competitors (IgG2a-isotype control, IgG2b-isotype control, 91R, or 92R) and revealed with peroxidase conjugated streptavidin. ***P < 0.001.

Furthermore, competition analyses demonstrated that 92R competes with itself and 91R for binding to the CCR9^+^ cells MOLT-4, but not with the 3C3 anti-hCCR9 mAb in flow cytometry. Similarly, 91R competes with itself and 92R but not with 3C3 mAb, as demonstrated by flow cytometry (Figure 2A). These data were corroborated by ELISA assays, where binding of biotinylated 91R to the hCCR9 (aa 2–22) synthetic peptide was competed by unlabeled 92R, but not by the isotype control antibody IgG2a. Similarly, binding of biotinylated 92R to the same peptide was competed by unlabeled 91R, but not by the isotype control antibody IgG2b (Figure 2B).

**Figure 3:**
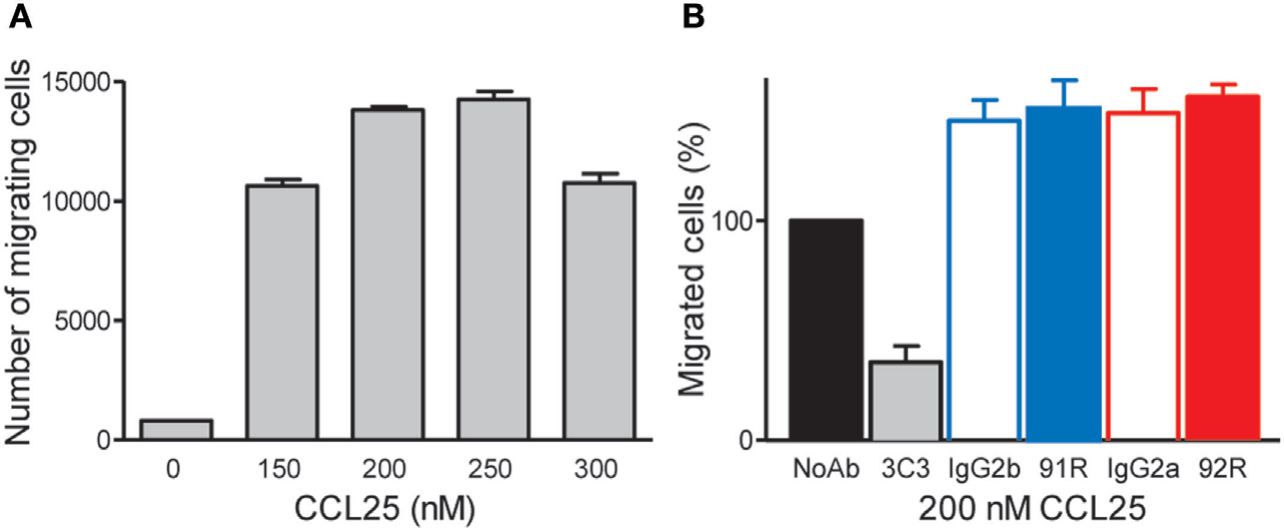
92R and 91R mAbs failed to inhibit CCL25-mediated migration of the CCR9^+^ MOLT-4 cells. **(A)** Migration response of MOLT-4 cells to different CCL25 concentrations. **(B)** 200 nM CCL25, in the absence of antibody was used to determine the highest number of migrating cells (defined as 100% migration) and to compare with similar experiments where 91R, 92R, or their isotype control antibodies (IgG2g or IgG2a, respectively) were added. In addition, the anti-CCR9 mAb 3C3, known to inhibit the CCL25:CCR9 interaction was used as positive control for migration inhibition.

In addition, 92R fails, similarly to 91R (34), to inhibit CCL25-induced migration of CCR9^+^ MOLT-4 cells, unlike the anti-CCR9 mAb 3C3, described to inhibit the CCL25:CCR9 interaction. Indeed, we determined the migration response of MOLT-4 cells to different CCL25 concentrations, observing that it reached the maximum level between 200 and 250 nM CCL25 (Figure 3A). Then, using 200 nM CCL25, the percentage of migrating cells in the absence or presence of different antibodies anti-hCCR9 or their isotype controls was determined. The results show a migration inhibition only in the presence of 3C3 mAb, but neither 92R nor 91R (nor their isotype controls) inhibited this migration (Figure 3B).

### Identification of the Critical aa for the Binding to the hCCR9 Epitope by 92R and 91R mAbs

**Figure 4:**
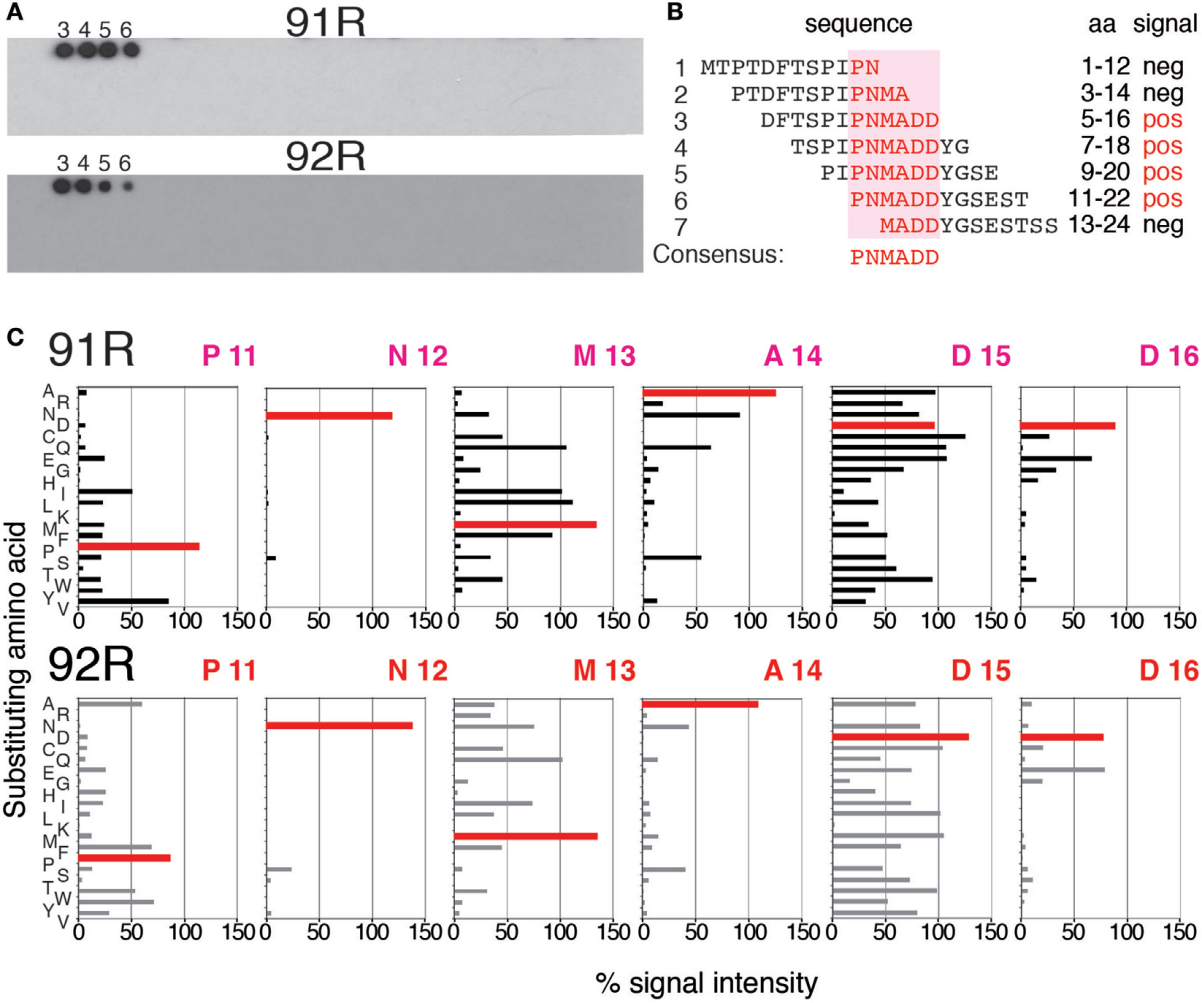
Identification and characterization of the energetically critical amino acid (aa) sequence recognized by 91R and 92R. **(A)** Pepscan analyses were used to identify the hotspots recognized by 91R and 92R mAbs. One hundred and eighty overlapping peptides, 12 aa long each, covering the full sequence of the hCCR9-A isoform, were synthesized on a cellulose membrane. After blocking, the membrane was incubated with 91R or 92R mAbs. Binding of the antibodies was revealed with a peroxidase-coupled anti-mouse IgG antibody and ECL (*n* = 2). **(B)** The sequence of peptides 1–7, including the four giving positive signals for 91R and 92R mAbs are aligned, and the minimum sequence recognized described as it was the unique entire sequence present in all of these peptides (aa 11–16). **(C)** Densitometric quantification of pepscan assays where aa from positions 11–16 were individually substituted by the other 19 proteinogenic aa after binding to 91R or 92R.

**Figure 5:**
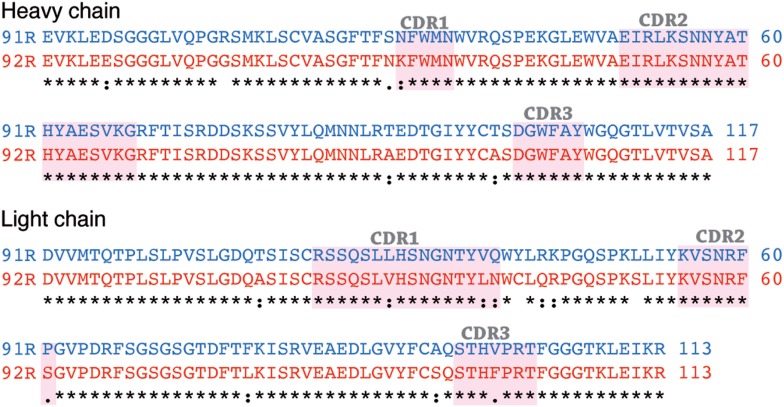
Comparison of amino acid sequences of light and heavy chain variable regions of 91R and 92R. Alignment of light and heavy chain variable IgG regions using the Clustal program version 2.1 The CDR determinants, identified following Kabat’s model are shaded. There are differences between 91R and 92R sequences both in the framework region and in the CDR determinants.

Since 92R and 91R are able to cross-compete with each other for the binding of hCCR9 on the cell surface (Figure 2A), and both of them bind the same epitope, comprised by aa 2–22 of hCCR9-A (Figure 2B), we aimed to identify the energetically critical aa for the interaction “hotspots” within the epitope. For this reason, pepscan analyses were carried out using 180 overlapping synthetic peptides, covering the entire hCCR9 sequence. These experiments allowed to identify the hotspot residues within the epitope, energetically critical for high-affinity binding of each antibody. Indeed, both antibodies gave positive signals with the same peptides 3–6 (Figure 4A), allowing to identify aa 11–16 from hCCR9-A as the functionally critical residues on the epitope recognized by 92R and 91R (Figure 4B). It is worth to note that although the peptides identified by these mAb are the same, the signals obtained with 91R and 92R are somehow different from each other (Figure 4A). Subsequently, each one of the aa from hotspots (sequence PNMADD) was substituted for one of the 19 other aa, allowing to ascertain the relative relevance of each one of the energetically critical aa for the binding of 91R and 92R to the hCCR9 synthetic peptides (Figure 4C). For both 91R and 92R mAbs, turned out that in these assays, the binding of these antibodies to the hotspots are strictly dependent of the presence of an N residue in position 12. The only replacement allowed at position 16 is a D for an E, indicating the relevance of a negative charge at this position. A14 could be replaced by hydrophilic uncharged aa (N, Q, or S) with a significant reduction of signal intensity. The few allowed changes for P11, unlike for A14, are more evident for 91R. Conversely, M13 and D15 allow more changes, with appreciable signal differences between 92R and 91R. For both mAb, the substitution of D15 for K or P is not allowed. The substitution of D15 for R is allowed for 91R but 92R loses its binding capacity. Taken together, these data support the notion that 91R and 92R are different antibodies, since the allowed changes on each position were different. This was further corroborated by sequence analyses of the variable regions from the light and heavy chain cDNAs (Figure 5). Indeed, there are five aa differences on the heavy chain framework and ten on the light chain framework. In addition, on the heavy chain, there is an aa change in CDR1, and on the light chain, there are two aa changes in CDR1, one in CDR2 and another one in CDR3 (Figure 5).

**Figure 6:**
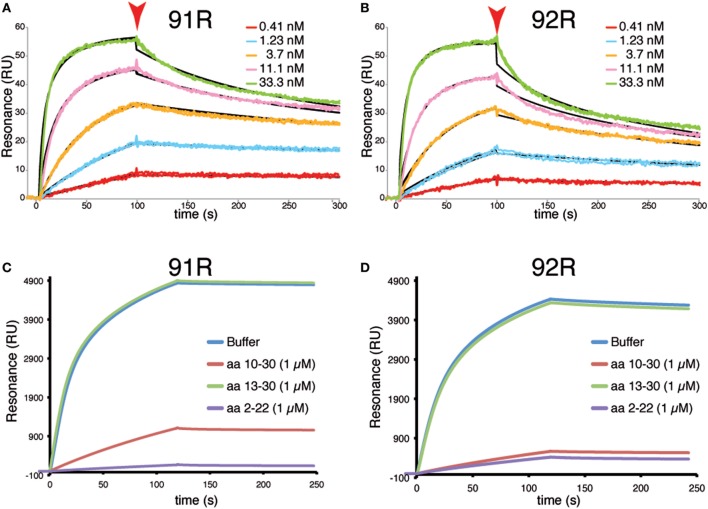
Surface plasmon resonance analyses of the interaction between 92R or 91R and hCCR9 synthetic peptides. A synthetic peptide corresponding to hCCR9A aa 2–22 was immobilized on a carboxymethyl-dextrane CM5 chip by an amino coupling method. Afterward, different concentrations of 91R **(A)** or 92R **(B)** mAb were added, ranging from 0.41 to 33 nM. The black sensograms show the fitted curves obtained with the Biaevaluation 4 software. Competitive experiments were analyzed by binding of 91R **(C)** or 92R **(D)** to a biotinylated synthetic peptide hCCR9(2–19)-captured onto the surface of a SA sensor chip. As competitors, 1 µM of peptides hCCR9(2–22), hCCR9(10–30) and hCCR9(13–30) were used. The differences between experimental and control flow cells is given in resonance units (RU).

Surface Plasmon resonance analyses using the synthetic surface-bound peptide hCCR9 (2–22) as molecule representative of CCR9, allowed to determine the apparent affinity constants for these antibodies, which was estimated as *K*_D_ 3.6 nM for 91R and *K*_D_ 8.9 nM for 92R (Figures 6A,B). Competition experiments showed that 1 µM of peptide hCCR9 (2–22) inhibited most of the binding of 92R to the surface and fully inhibited the 91R binding; whereas peptide hCCR9 (13–30), corresponding to the N-terminus of the B isoform, failed to inhibit mAb binding. The competition with the same concentration of the peptide hCCR9 (10–30), which also contains the hotspots, gave, however, a slightly different competition pattern between 92R and 91R, since for 92R, the sensograms were similar when the competitors were hCCR9(10–30) or hCCR9(2–22), whereas a 20% of the 91R signal was still detected at the end of the association phase in the presence of hCCR9(10–30) peptide (Figures 6C,D). Taken together, these data show that the two antibodies 91R and 92R are able to bind differentially to the same hCCR9 epitope with high affinity.

### 92R mAb Inhibits *In Vivo* the Growth of Human CCR9^+^-Tumors in Xenografts

**Figure 7:**
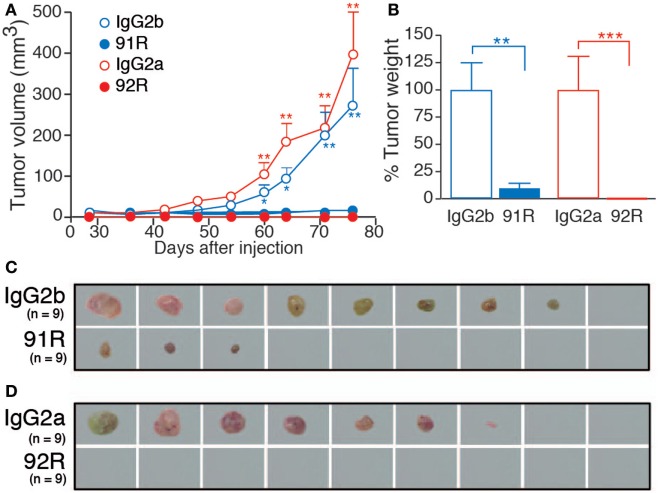
Leukemia xenograft growth is reduced in mice treated with 92R or 91R mAb. For xenograft analyses, MOLT-4 cells (2 × 10^6^) were inoculated subcutaneously in Rag2^−/−^ mice on day 0. Experimental groups received four intraperitoneal doses of 91R, 92R, or their isotype-controls (irrelevant IgG2b and IgG2a mAbs, respectively). First and second inoculations were with 4 mg/kg; whereas third and fourth inoculations were with 2 mg/kg. Tumor growth was measured with a Vernier caliper every 3 days. After mice were sacrificed on day 78, tumors were removed and weighed. **(A)** Tumor growth kinetics. Tumor volume was measured at times indicated and calculated as *V* = [axial diameter length, mm] × [(rotational diameter, mm)^2^/2] (9 mice/group). **(B)** Tumor weight (%) relative to IgG2b (or IgG2a) treatment on day 78. Mean ± SEM (*n* = 9 mice/group). **(C)** Images of tumors from IgG2b-isotype control or 91R-treated mice at the time of sacrifice (day 78). **(D)** Images of tumors from IgG2a-isotype control or 92R-treated mice at the time of sacrifice (day 78). ***P < 0.001, **P < 0.01, *P < 0.05.

The antitumor potential of 92R mAb was assessed in immunodeficient (Rag2^−/−^, BALB/c) mice, after subcutaneous injection of 2 × 10^6^ CCR9^+^ cells from the human T-ALL cell line MOLT-4. On days 1, 8, 15, and 22 after tumor cell injection, the animals were treated with either 92R, 91R, or their isotype controls (IgG2a or IgG2b, respectively) at 4 mg/kg (days 1 and 8), or 2 mg/kg (days 15 and 22). The size of developing tumors was measured until day 78, when mice were sacrificed. The differences between tumor size were significant on 91R-treated mice as compared to isotype-treated mice from day 60 (*P* < 0.05) (Figures 7A,C). On 92R, we failed to detect tumors, although they grew in their control isotype-treated mice (Figures 7A,D). At the time of sacrifice, tumors were removed and weighted. The mean tumor weight for IgG2b isotype-treated control was 1.23 ± 0.33 g and for IgG2a, isotype-treated control was 1.25 ± 0.39 g, whereas for 91R-treated mice was 0.11 ± 0.06 g (Figure 7B) and were absent in animals treated with 92R (Figures 7B,D). As a positive control, 91R was used, where we found a reduction of 91% on tumor burden, in agreement with previously published data (34). As mentioned above, for 92R, no tumors were detected after sacrificing the animals, although 6/9 animals treated with its isotype control (IgG2a) had tumors (Figure 7D). These data support that 92R efficiently blocked the *in vivo* progression of acute T cell leukemia xenografts and suggest the possibility of using this antibody for therapeutic purposes in human CCR9^+^ tumors.

### 92R mAb Mediates Complement-Dependent and Antibody-Dependent NK Cell-Mediated Cytotoxicity

Complement-dependent cytotoxicity and ADCC are two of the main mechanisms *in vivo* for tumor cell elimination by therapeutic antibodies. For this reason, we tested the *in vitro* ability of 92R to induce lysis of MOLT-4 leukemia cells by either complement fixation, or by NK cell mediated cell cytotoxicity triggered by binding of 92R mAb to the NK cell surface Fc receptors.

**Figure 8:**
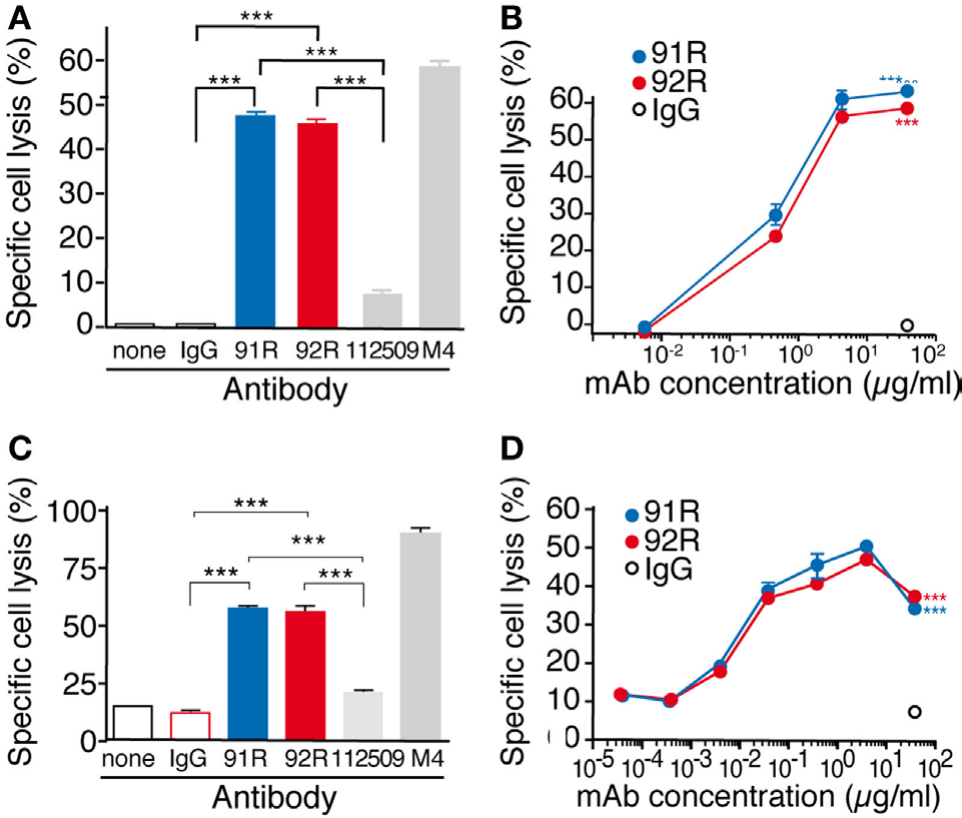
92R promotes complement-dependent cytotoxicity and antibody-dependent cell-mediated cytotoxicity in MOLT-4 human leukemic cells. **(A)** MOLT-4 cells were opsonized with 92R, 91R, or isotype-matched mAb (40 µg/ml, 30 min, 37°C), washed, and incubated (1 h) with 25% active (37°C) or inactive (56°C) baby rabbit complement; cell viability was evaluated in a flow cytometer after 7-AAD staining. Specific complement lysis in the absence of antibody or with 91R, 92R, 112509, M4 or isotype-matched mAb (IgG2a or IgG2b). Each condition was analyzed in triplicate. Data show mean ± SEM of four independent experiments. **(B)** Dose–response curve for specific complement lysis using 91R or 92R and a control IgG2b mAb at indicated concentrations. Data show mean ± SEM of a representative experiment. **(C)** Specific NK-dependent cytotoxicity mediated by 91R, 92R, 112509, isotype-matched mAb (IgG2a or IgG2b) or positive control M4 serum. NK cells were isolated from BALB/c spleens and cultured for 6–7 days in medium containing mrIL-2. CFSE-labeled MOLT-4 target cells were preincubated with by 91R, 92R, 112509, isotype-matched mAb (IgG2a or IgG2b), or positive control M4 pooled sera (1:1,000) (30 min, 37°C). NK cells and labeled target cells were then cocultured at a 20:1 ratio (4 h, 37°C). Specific lysis was determined by staining dead cells with 7-AAD and analyzing the number of 7-AAD^+^ green cells by flow cytometry. Each condition was analyzed in triplicate. Data show mean ± SEM (*n* = 5 independent experiments). **(D)** Dose–response curve for specific complement lysis using 91R and a control IgG2b mAb at indicated concentrations. Data show mean ± SEM for duplicates from one representative experiment of four. ***P < 0.001, **P < 0.01, *P < 0.05.

For the CDC experiments, specific death of the MOLT-4 cells was evaluated by flow cytometry analyses of 7-AAD incorporation. Both 92R and 91R were able to promote complement-dependent cell lysis (46 ± 1 and 49 ± 2%, respectively) with a much higher efficiency than the commercial anti-CCR9 antibody 112509 (7.1 ± 0.9%) (Figures 8A,B). As positive control, we used a mouse sera (M4) against MOLT-4, which gave a specific lysis of 60 ± 0.8%. The minimal concentration of 92R mAb that gave a detectable specific cell lysis was 0.4 µg/ml.

For ADCC experiments, MOLT-4 target cells labeled with the green fluorescent dye CFSE and precoated with either 92R, or isotype control (IgG2a, negative control) were combined with previously *in vitro* activated mouse NK cells (4 h at 37°C). The incorporation of 7-AAD by the CFSE-labeled cells allowed to determine specific NK-mediated killing of MOLT-4 cells by flow cytometry. On these experiments, 91R mAb was used as positive control. Both 92R and 91R were able to promote NK-dependent cell lysis of the cells (57.7 ± 1.1 and 54.9 ± 4.1, respectively) with a much higher efficiency than the commercial anti-CCR9 antibody 112509 used as a control (20.5 ± 0.9%) (Figures 8C,D). As an additional positive control, we used the mouse serum M4 raised against MOLT-4, which gave a specific lysis of 91.1 ± 1.1%. The 92R mAb concentration needed for a detectable response was 0.04 µg/ml, whereas it was negligible in the absence of antibody. 91R was used as positive control for ADCC, obtaining similar results to the previously published (34).

**Figure 9:**
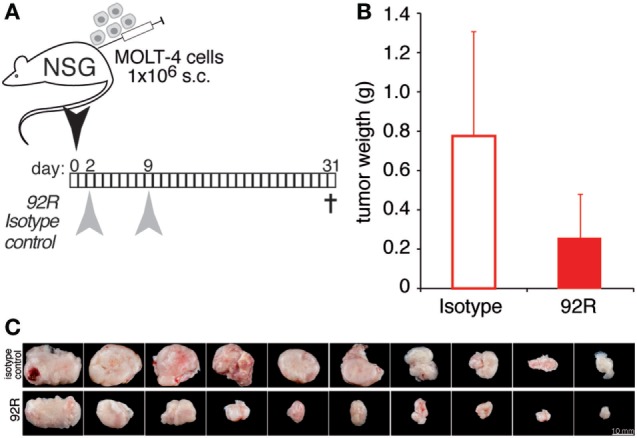
92R mAB reduces leukemia xenograft growth in NSG. MOLT-4 cells (1 × 10^6^) were inoculated subcutaneously in NSG mice on day 0. Experimental groups received two intraperitoneal doses of 92R or its isotype-control (IgG2a mAb). **(A)** antibody administration schedule, the animals received 4 mg/kg each on days 2 and 9 of either 92R or the control isotype IgG2a mAb. Mice were sacrificed on day 31, when tumors were removed and weighed. **(B)** Tumor weight in grams of IgG2a (isotype control) and 92R treated animals on day 31. Mean ± SEM (*n* = 10 mice/group). **(C)** Images of tumors from IgG2a-isotype control or 92R-treated mice at the time of sacrifice (day 78).

Taken together, these results suggest a role for CDC and ADCC in the *in vivo* reduction of tumor growth observed in the xenograft model. To directly determine the *in vivo* role of CDC and ADCC in the antitumoral potential of 92R mAb, we generated subcutaneous xenografts by injection of 1 × 10^6^ CCR9^+^ cells from the human T-ALL cell line MOLT-4 in NSG mice. We used this particular mouse strain because its complement is not functional (due to a mutation in the C5 gene), and their NK activity is highly diminished (Jackson Labs, Bar Harbor, MD, USA). Therefore, if 92R was able to inhibit tumor growth in this model, the mechanism(s) of *in vivo* tumor reduction would be different from CDC and ADCC. On days 2 and 9 after tumor cell injection, the animals were treated with either 92R or its isotype control (IgG2a) at 4 mg/kg. On day 31, mice were sacrificed (Figure 9A), tumors removed, and weighted. The mean weight of the tumors was smaller in 92R-treated mice (0.256 ± 0.22 g) as compared to control isotype-treated mice (0.776 ± 0.53 g) (Figure 9B); therefore, the total tumor burden, measured as the mean of the tumor weights for each group, was reduced by 67% on animals treated with 92R, as compared to control isotype-treated animals, which correlated with the pictures taken of the tumors (Figure 9C). These data support the notion that mechanisms distinct from CDC and ADCC may play a role on the inhibition of acute leukemia tumor growth mediated by 92R *in vivo* in xenografts generated by human CCR9^+^ tumors. However, *in vitro* experiments analyzing whether 92R could mediate the inhibition of tumor growth through apoptosis (Figure S1 in Supplementary Material) or phagocytosis (Figure S2 in Supplementary Material) gave negative results.

## Discussion

Patients affected of human T-lineage acute lymphoblastic leukemia (T-ALL) undergo chemotherapeutic treatment; however, a significant amount of them relapse or are refractory to the standard treatment and then should undergo bone-marrow transplantation subsequent to whole body irradiation, with uncertain results (the 5-year overall survival for young adults and adolescents is around 50%, while for children it is near 80%) (38, 39). Therefore, a less invasive therapy would be beneficial for these patients. We believe that the use of mAb-based therapies, as they are being used for other cancer types, would be a real improvement for them.

Overexpression of homeostatic chemokine receptors in tumor cells is linked to cancer progression, metastasis, and poor prognosis (22). Many reports describe a relevant role for the CCL25/CCR9 axis in cancer progression (26, 30), in particular, CCR9 expression has been associated with leukemia aggressiveness (17), its aberrant expression has been detected in several solid tumors (14, 15, 17–30, 40) and has been associated in organ selective metastasis of melanoma to small intestine (14–16), suggesting its potential as a target for cancer treatment. In this context, we have described the generation and characterization of mAb 91R, which inhibits CCR9^+^-tumor growth on *in vivo* subcutaneous xenografts of human ALL in immunodeficient Rag2^−/−^ mice. We continued this work to select other anti-CCR9 mAb with different epitope specificities, affinities, mechanisms of action and if possible, with higher melting points.

Here, we report the identification and characterization of 92R mAb, an anti-hCCR9 antibody that was raised using as immunogen the full cDNA sequence of the human CCR9-A receptor, which is the main expressed isoform (41). As expected, 92R identifies hCCR9-transfected cells and endogenous CCR9 expressed in MOLT-4 cells. The observation that 92R and 91R cross-compete with each other for binding to the antigen in flow cytometry assays suggests that 92R recognizes an epitope present in the hCCR9 N-terminal domain. This assumption was confirmed by the ELISA data where 92R was able to bind to the synthetic peptide aa 2–22 of hCCR9-A. The energetically critical residues within the hCCR9 epitope required for 92R binding were determined by Pepscan, where a set of overlapping peptides that span all the CCR9-A aa sequence was used. Both 92R and 91R mAb identified as hotspots the aa sequence 11–16 of the receptor, although, the relative intensity signals for each of the recognized peptides was different. Strongly suggesting that 92R and 91R mAb bind differentially to the epitope. Fine mapping of the hotspot sequence shows the relevance of each of these aa, where N12 is strictly required for the interaction with both mAb (92R and 91R). However, the replacement of D15 for R is allowed for 91R, but not for 92R, and the few allowed changes for P11 are distinct for 91R and 92R. Taken together, these data suggest that 92R and 91R are two different antibodies. This was corroborated by aa sequence analysis of the variable heavy and light chains of these antibodies.

Interestingly, on the heavy chain hyper-variable regions (CDR), there is only one aa difference between both mAb. In CDR1, 92R has a positive charged aa (K) that might justify the lack of interaction with the peptide when the negatively charged D15 is replaced by R. In CDR2, most of the aa are hydrophilic, and many of them charged, with a balance of positive charged aa. This suggests that the electrostatic interactions between these antibodies with CCR9 are very important. Furthermore, in the light chain CDRs, most of the aa differences between 92R and 91R are conservative, and do not seem to be responsible for the signal patterns observed in the Pepscan analyses.

SPR data shows that for both 92R and 91R, the apparent *K*_D_ of their interaction with the peptide hCCR9(2–22) are on the nM range, although it is very likely that their affinities for hCCR9 are higher due to the absence in the synthetic peptide of the posttranslational modifications described for CCR9 and other chemokine receptors, such as sulfation or glycosylation (34, 42–44). Furthermore, SPR competition experiments demonstrate that these mAbs most likely do not identify the hCCR9-B isoform that starts in M13 of hCCR9-A, in full agreement with the lack of signal with peptide in position 7 on the Pepscan membranes. Moreover, 91R is able to discriminate between peptides hCCR9(2–22) and hCCR9(10–30) but, 92R does not. This implies that aa 2–9 from the hCCR9-A isoform, contained within the epitope recognized by these mAb, but outside from the defined hotspot sequences (aa 11–16) might also be required for the high affinity binding of 91R, but not for 92R, strengthening the notion that there are functional differences between these two mAb.

The functional differences between 92R and 91R were corroborated by the results of *in vivo* inhibition analyses of tumor growth in xenograft models, where in 91R-treated animals, 3 out of 9 developed tumors, whereas no tumors were detected on 92R-treated animals. We could not exclude the possibility that these differences were due to the different isotypes of the mAb (IgG2b for 91R, IgG2a for 92R). However, both antibodies were able to elicit CDC and ADCC *in vitro* against CCR9^+^ cells, without significant quantitative differences. To further dissect the mechanism(s) involved in 92R-mediated tumor growth inhibition, and to determine the *in vivo* relevance of CDC and ADCC, another strain of immunocompromised mice was used, characterized by an impaired complement and NK cell activities, in addition to lack of T and B lymphocytes (NSG mice). The data obtained in the experiments using NSG mice show a fundamental role for CDC and ADCC as 92R *in vivo* mechanisms of action, since unlike on the experiments carried-out in Rag2^−/−^ mice, in this case, all the 92R-treated animals (*n* = 10) had tumors. However, it also points out that 92R can inhibit tumor growth through additional mechanisms. Indeed, 92R is able to reduce 67% the tumor burden in animals with impaired NK and complement activities. These additional mechanisms are not yet unraveled, and despite the *in vitro* data suggesting that apoptosis or phagocytosis do not play a role, we cannot formally exclude that 92R might use these mechanisms *in vivo* to inhibit MOLT-4 tumor growth.

Taken together, the data presented here suggests an antitumoral potential for 92R mAb, since in addition to its ability to inhibit tumor growth in xenografts, is able to kill the tumor cells through multiple mechanisms of action, making it an excellent therapeutic agent candidate against CCR9^+^-tumors.

## Ethics Statement

Animal care and treatment were carried out in accordance with Spanish and EU laws. The CSIC Ethics Committee approved these experiments and the Community of Madrid Agriculture Department approved the use of experimental animals: PROEX 038/17 (to JG-S) and PROEX 164/16, PROEX 17/14 and PROEX 121/16 (to LK).

## Author Contributions

LK designed the immunizations, carried out the cell fusions, and the initial screening. BS-C, IC-G, and MV carried out the *in vitro* and *in vivo* experiments with Rag2^−/−^ mice. MM carried out the competition assays and affinity measurements with the Biacore biosensor, SS carried out the *in vivo* experiments with the NSG mice. JG-S and LK were responsible for the overall concept and design of the study, data interpretation, and writing, together with BS-C the final manuscript. All authors contributed to drafting, revising, and approving the final article.

## Conflict of Interest Statement

The authors declare that the research was conducted in the absence of any commercial or financial relationships that could be considered as a potential conflict of interest. JG-S and LK are inventors of a patent application covering 91R and 92R mAbs, owned by the CSIC.
