# Supplementary material for: 92R Monoclonal Antibody Inhibits Human CCR9+ Leukemia Cells Growth in NSG Mice Xenografts
Source: Front Immunol. 2018 Jan 29;9:77. doi: 10.3389/fimmu.2018.00077 (PMC5797297; doi:10.3389/fimmu.2018.00077)
Supplement: Supplementary file 1 [file Image_1.PDF]

Somovilla-Crespo, B., Martín Monzón, M.T., Vela, M., Corraliza-Gorjón, I., Santamaria, S., Garcia-Sanz, J.A. and Kremer, L. 92R monoclonal antibody inhibits human CCR9+ leukemia cells growth in NSG mice xenografts. Front. Immunol. 9:77. Doi: 10.3389/fimmu.2018.00077

Supplementary Figure 1

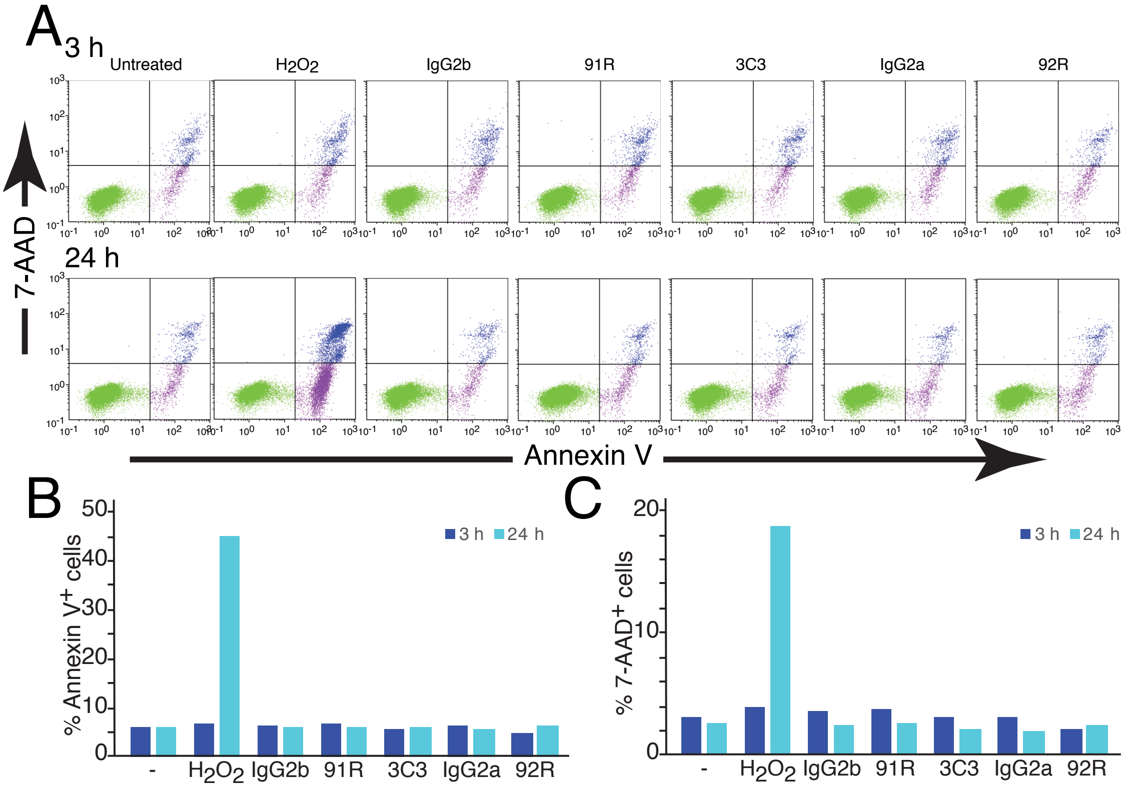

Supplementary Figure 1. 92R mAb fails to induce apoptosis in MOLT-4 cells. (A) FACS analysis of MOLT-4 cells stained with Annexin V-FITC and 7-AAD after culture for 3 h or 24 h with the anti-hCCR9 mAbs 91R, 3C3 or 92R, to determine the fraction of apoptotic and necrotic cells. As negative controls, cells treated with isotypic antibodies (IgG2b or IgG2a) were used. As a positive control, the cells were treated with H_2O_2. (B) Quantification of the fraction of cells positive for Annexin V. (C) Quantification of the fraction of cells positive for 7-AAD. The data shown is from a representative experiment out of three.

Methods: MOLT-4 cells ($5x10^5$ cells/ml, 96% of the cells excluded Trypan blue) were cultured in Dulbecco's modified Eagle's medium supplemented with 10% FBS, 2 mM L-glutamine, 50 U/ml penicillin, and 50 µg/ml streptomycin. The cells were incubated with complete medium alone or containing either H_2O_2 (0.2 mM) or mAb (20 µg/ml). At the indicated times, cells were collected, washed 2 times with PBS and incubated with Annexin V-FITC (SouthernBiotech) and 7-AAD (BD Biosciences), and analyzed on an Epics XL or a Cytomics cytometer (Beckman Coulter).
