# Supplementary material for: 92R Monoclonal Antibody Inhibits Human CCR9+ Leukemia Cells Growth in NSG Mice Xenografts
Source: Front Immunol. 2018 Jan 29;9:77. doi: 10.3389/fimmu.2018.00077 (PMC5797297; doi:10.3389/fimmu.2018.00077)
Supplement: Supplementary file 2 [file Image_2.PDF]

Somovilla-Crespo, B., Martín Monzón, M.T., Vela, M., Corraliza-Gorjón, I., Santamaria, S., Garcia-Sanz, J.A. and Kremer, L. 92R monoclonal antibody inhibits human CCR9+ leukemia cells growth in NSG mice xenografts. Front. Immunol. 9:77. Doi: 10.3389/fimmu.2018.00077

Supplementary Figure 2

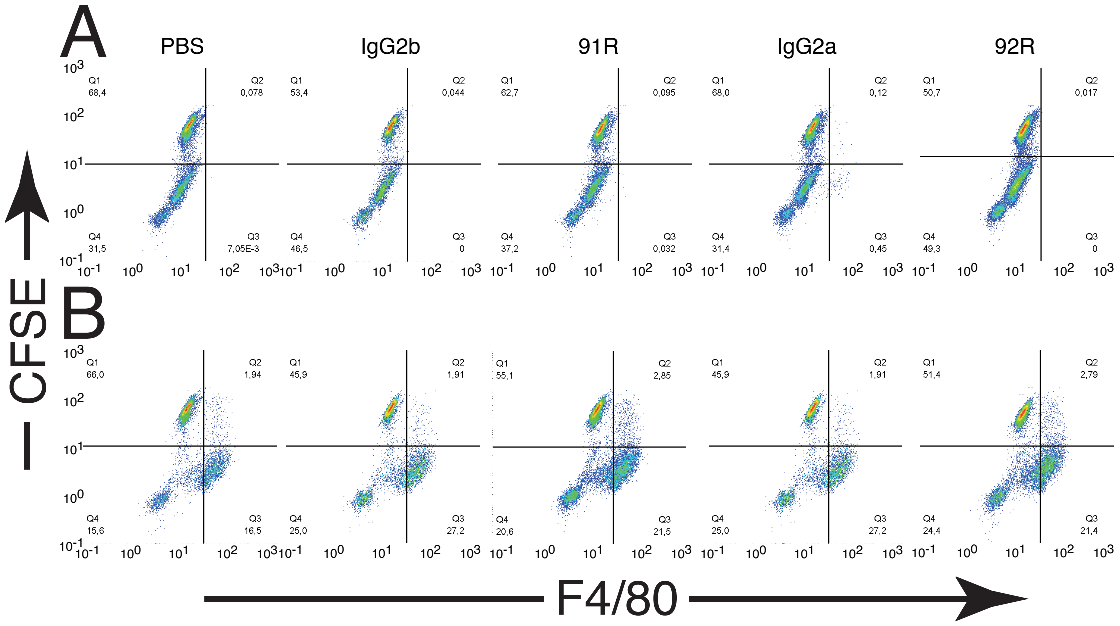

Supplementary Figure 2. 92R fails to induce in vitro phagocytosis on MOLT-4 cells. Thioglycollate-elicited mouse peritoneal macrophages were co-cultured with CFSE-labelled MOLT-4 cells in the presence of 91R, 92R or the isotypic antibodies as controls. After 4 h incubation, cells were stained with F4/80 mAb. Direct interactions between the opsonized-MOLT-4 cells and the macrophages can be seen as double positive dots, macrophages alone as single positive dots (red) and MOLT-4 cells alone as single positive dots (green) (B). As control, the co-cultures containing the opsonized-MOLT-4 cells and the macrophages were analyzed without staining with F4/80 (A).

Methods: Thioglycollate-elicited peritoneal exudate cells from Rag2$^{-/-}$ BALB/c mice were used to ascertain phagocytosis of 91R or 92R (50 μg/ml) opsonized CFSE-labelled MOLT-4 cells (1hr, 4ºC). After 4h co-incubation of the peritoneal exudate cells and the opsonized MOLT-4 cells, the co-cultures were stained (or not) with F4/80 mAb, fixed and analyzed by flow cytometry.
